# Combined Interhemispheric and Endoscopic Endonasal Resection of a Rare Olfactory Schwannoma with Preservation of Olfactory Function

**DOI:** 10.3390/curroncol33090515

**Published:** 2026-08-28

**Authors:** Leonardo Anselmi, Alexandre Lavé, Kristof Egervari, Basile N. Landis, Philippe Bijlenga, Julien W. Hsieh, Paul E. Constanthin

**Affiliations:** 1 Division of Neurosurgery, Department of Clinical Neurosciences, Geneva University Hospitals, University of Geneva, 1205 Geneva, Switzerland; 2 Department of Neurosurgery, IRCCS Humanitas Research Hospital, 20089 Milan, Italy; 3 Faculty of Medicine, University of Geneva, 1202 Geneva, Switzerland; 4 Division of Neuropathology, Department of Clinical Pathology, Geneva University Hospitals, 1211 Geneva, Switzerland; 5 Rhinology-Olfactory Unit, Department of Otorhinolaryngology—Head and Neck Surgery, Geneva University Hospitals, 1211 Geneva, Switzerland

**Keywords:** olfactory schwannoma, fronto-ethmoidal tumor, combined surgical approach, interhemispheric resection, endoscopic endonasal surgery, rare intracranial tumor

## Abstract

Introduction: Olfactory groove schwannomas (OGSs) are exceptionally rare intracranial tumors, with fewer than 80 cases reported. Their imaging features often mimic meningiomas or esthesioneuroblastomas, complicating preoperative diagnosis. Their origin remains debated due to the absence of Schwann cells in the olfactory nerve. Research Question: To describe a rare case of olfactory groove schwannoma with ethmoidal extension, successfully treated through a combined interhemispheric and endoscopic endonasal approach, and to discuss its diagnostic and surgical implications in light of the current literature. Furthermore, to measure the respective olfactory function before and after surgery. Material and Methods: A 52-year-old man presented with transient visual disturbances, headache, lexical access difficulties, slight executive dysfunction with impaired inhibition, and anterograde verbal memory impairment. He had no olfactory complaints but olfactory testing revealed unilateral, left-sided anosmia. Preoperative MRI demonstrated a large left olfactory groove mass with solid–cystic components, bone erosion, and inferior ethmoidal extension. A combined transcranial interhemispheric and endoscopic endonasal approach was performed to achieve total resection and ensure skull base reconstruction. Results: Gross total tumor removal was achieved. Histopathological examination confirmed a WHO grade I schwannoma. Postoperative recovery was uneventful, with improvement in neuropsychological and left-sided olfactory function. There was no residual lesion on follow-up MRI. Discussion and Conclusions: OGS should be included in the differential diagnosis of anterior skull base tumors with cystic or sinonasal extension. In selected cases, a combined cranio-endoscopic approach allows safe and radical resection while minimizing morbidity. Accurate histopathological evaluation remains essential for definitive diagnosis, as radiological features alone may be misleading. Olfactory function should be systematically measured since preservation and even improvement of olfactory function are possible. This supports a non-olfactory origin of the tumor, suggesting secondary compression rather than primary involvement of the olfactory system.

## Simple Summary

Olfactory groove schwannomas (OGSs) are exceptionally rare intracranial tumors representing a surgical challenge. We describe a case of OGS removed using a combined transcranial interhemispheric and endoscopic endonasal approach supported by the use of mixed reality to achieve total resection and ensure skull base reconstruction. Interestingly, postoperative testing revealed an improvement of the patient’s olfactory function.

## 1. Introduction

Schwannomas are benign nerve sheath tumors that arise from Schwann cells and most often involve the vestibular, trigeminal, or facial nerves [1]. Those arising at the olfactory groove are among the rarest, accounting for less than 1% of intracranial schwannomas [1], with fewer than 80 cases documented to date and almost all as isolated reports or small series [2]. Reported patients are usually adults, with a mean age of 32.7 years and a slight male preponderance [3,4,5,6,7,8]. Their occurrence is difficult to explain with the absence of Schwann cells along the olfactory nerve, and several competing theories have been put forward to explain where these tumors actually originate [3,4,5,6,7,8].

The clinical picture tends to be vague. Headache and a reduction or loss of smell are the usual presenting complaints, whereas visual symptoms emerge only when the tumor extends posteriorly and exerts mass effect on the optic apparatus [6,7]. One point stands out: despite involving the cranial nerve subserving smell, olfaction is typically recorded only in passing, if assessed at all, and rarely by any formal method [9].

Most of these lesions are solitary, unilateral, supratentorial masses located in the anterior cranial fossa. On imaging, these tumors usually appear as well-circumscribed, contrast-enhancing, extra-axial lesions in the anterior cranial fossa [6,10]. However, features such as heterogeneous enhancement, cystic components, bony erosion, and involvement of the nasal cavity or paranasal sinuses can mimic more common skull base tumors such as meningioma, sinonasal carcinoma or esthesioneuroblastoma [3,6,11,12]. Therefore, histopathology and immunohistochemistry remain essential for definitive diagnosis.

We present the case of a large OGS with ethmoidal extension and a significant intracranial cystic component, successfully treated via a combined interhemispheric and endoscopic endonasal approach. This case highlights the diagnostic ambiguity and surgical complexity of managing such uncommon anterior skull base tumors.

## 2. Illustrative Case

### 2.1. Clinical Presentation and Preoperative Imaging

A 52-year-old man with an unremarkable medical history came to attention because of transient visual disturbances that had been recurring over several months, accompanied by episodic headaches. He reported no nasal or olfactory complaints. Ten days before admission he developed an unusually severe headache, distinct from his previous episodes, which prompted emergency evaluation.

**Figure 1:**
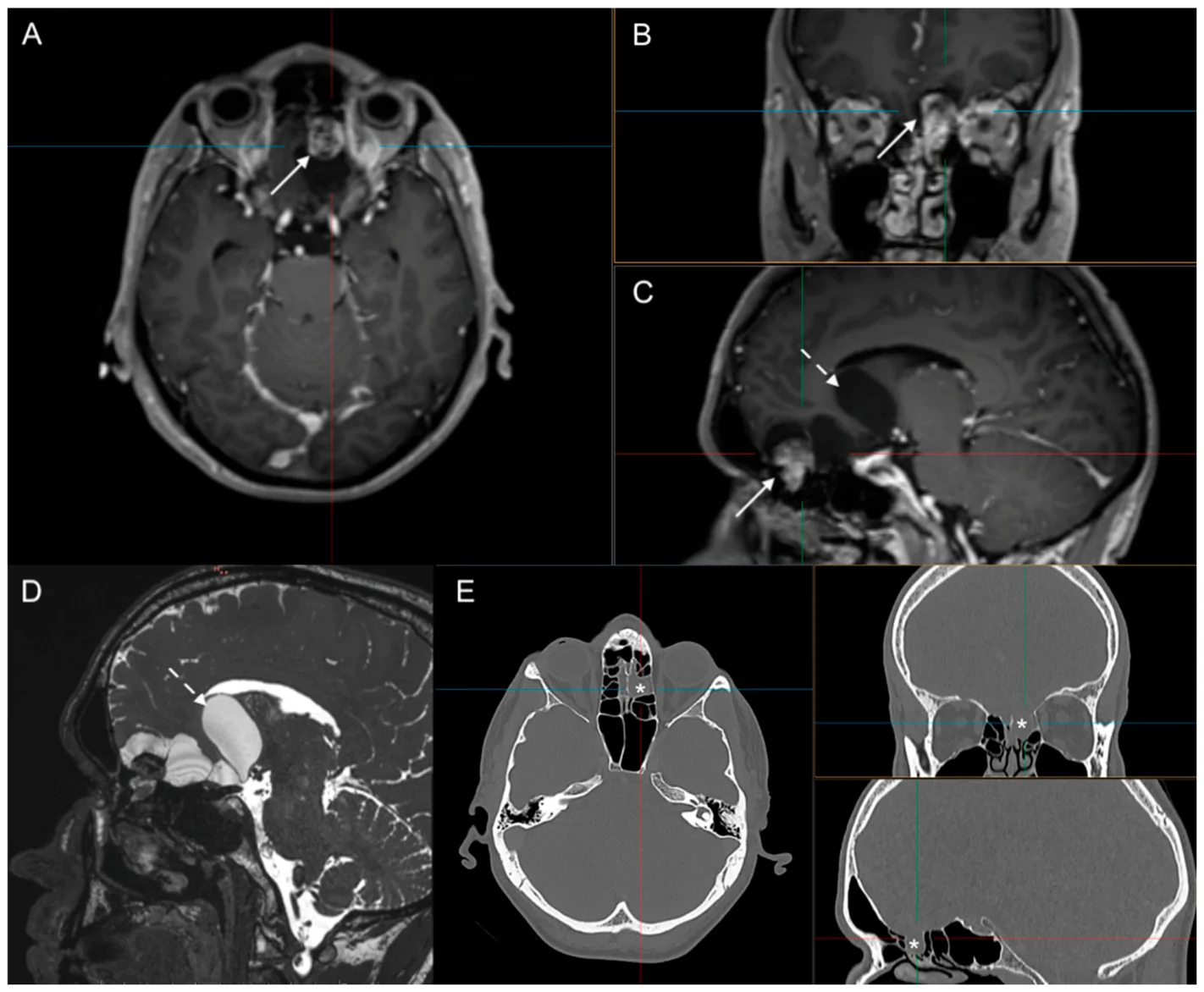
Preoperative imaging of the olfactory groove schwannoma, showing the solid component of the lesion (arrow), the cystic component (dashed arrow) and ethmoidal bone erosion (asterisk). (**A**) Axial T1-weighted post-contrast image MRI. (**B**) Coronal T1-weighted post-contrast MRI. (**C**) Sagittal T1-weighted post-contrast MRI. (**D**) Sagittal CISS sequence MRI. (**E**) Bone window CT scan.

MRI revealed a mixed solid–cystic mass centered on the left olfactory groove, extending into the ethmoid sinus and nasal cavity, with mass effect on the optic apparatus and adjacent vessels. The solid portion enhanced heterogeneously and contained hemorrhagic foci, whereas the large frontal cyst showed no peripheral enhancement. Medially the lesion reached the crista galli and laterally the lamina papyracea; inferiorly it extended into the nasal cavity, while its upper margin compressed the left optic nerve, optic chiasm, hypothalamus, and terminal segment of the internal carotid artery. CT scan confirmed ethmoidal invasion with bone erosion. Based on radiological features, a preliminary diagnosis of esthesioneuroblastoma was considered (Figure 1).

Lateralized olfactory testing revealed left-sided anosmia with a Sniffin’ Sticks composite TDI (threshold, discrimination, identification) score of 15 out of possible 48 points. The threshold test determines the lowest concentration of an odorant that can be perceived, with scores ranging from 1 (lowest) to 16 (best). The discrimination test assesses the ability to distinguish an odd odor from two identical odors across 16 triplets, while the identification test evaluates the ability to correctly identify 16 common odors by selecting the corresponding verbal descriptor from a set of choices. For discrimination and identification tests, the score is defined as the number of correct answers. Score details were: threshold = 1; discrimination = 3; and identification = 11. On the non-affected right side, the TDI score indicated normosmia (31.75—threshold = 7.75; discrimination = 11; identification = 13) [13]. The patient had no other cranial nerve deficits and no focal motor or sensory impairment. Nasal endoscopy was normal. Preoperative neuropsychological and speech–language assessment revealed mild lexical access weakness (naming at cut-off), slight executive dysfunction with impaired inhibition (Stroop task), and anterograde verbal memory impairment affecting both immediate and delayed recall. These findings were consistent with the tumor’s anatomical location.

### 2.2. Surgical Strategy and Technique

Surgical resection was performed using a combined approach to address both the intracranial and ethmoidal components of the lesion. The choice of approach followed directly from the tumor’s anatomy. Above, the lesion was in contact with the optic apparatus and the terminal internal carotid artery and carried a high frontal cyst—territory best reached through an interhemispheric corridor, which offers wide exposure of the anterior fossa with little need for brain retraction. Below, the tumor had eroded the ethmoid and extended into the sinonasal compartment, which called for endonasal resection and reconstruction. Since neither corridor could address both problems on its own, the two were combined.

**Figure 2:**
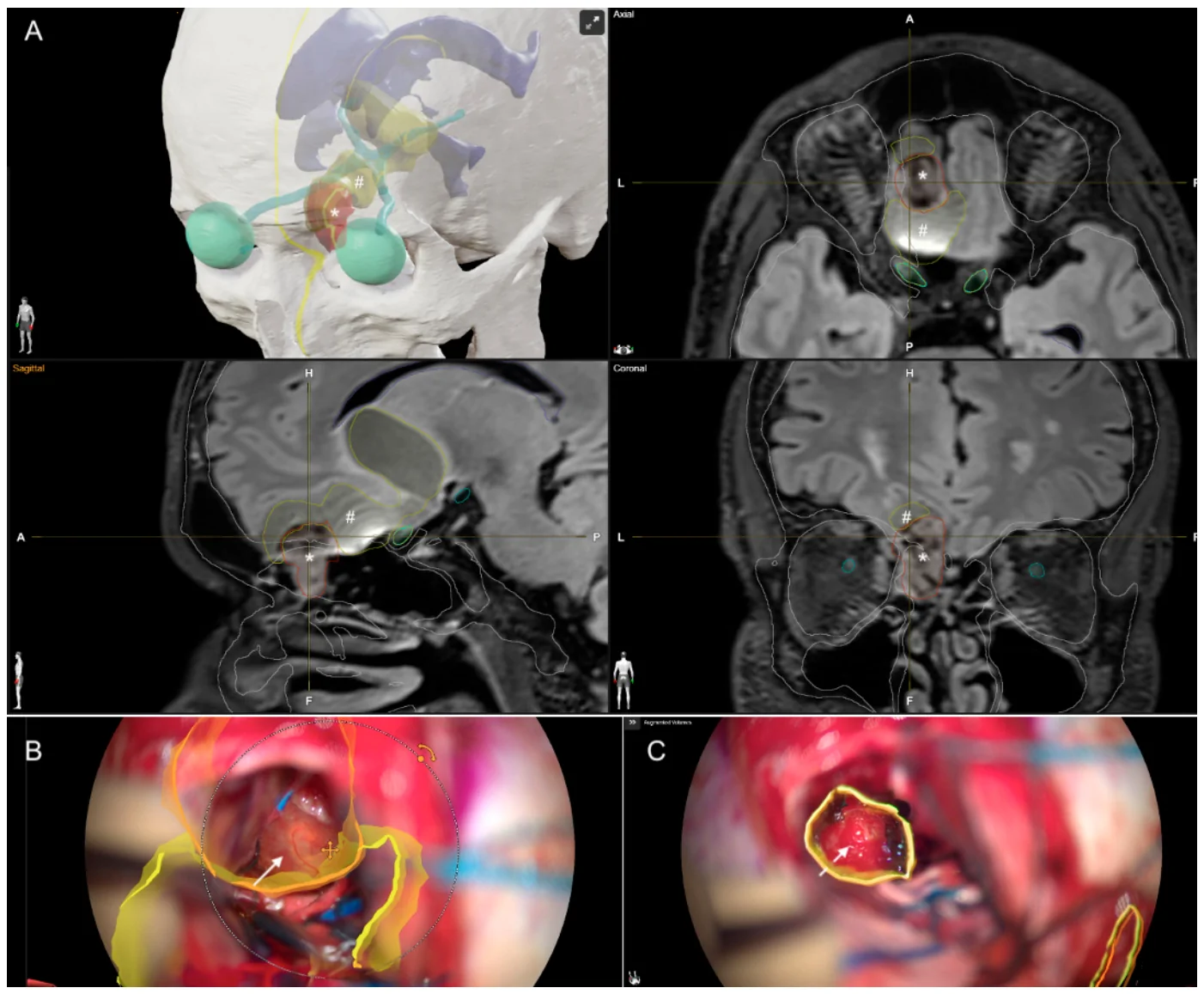
Intraoperative photograph demonstrating tumor resection and use of mixed reality overlay to delineate tumor margins. (**A**) Preoperative MRI with contrast in axial, coronal, and sagittal planes and 3D reconstruction from surgical planning highlighting the optic apparatus and delineating the solid (*) and cystic (#) components of the tumor. (**B**) Microscopic view with mixed reality overlay showing the solid portion of the tumor (arrow). (**C**) Microscopic view of the anterior cranial fossa following gross total resection of the lesion (dashed arrow). Anatomical orientation is indicated by the crosshair labels: A, anterior; P, posterior; H, head (superior); F, feet (inferior).

Neuronavigation and a mixed reality overlay (Brainlab) were used. The segmented models—comprising the solid and cystic tumor components, the optic apparatus, and the adjacent vessels—were overlaid onto the operating microscope view, so that these structures remained visible within the operative field, including before they were surgically exposed, without diverting attention to a separate navigation screen (Figure 2).

The patient was placed in dorsal decubitus under general anesthesia with the head fixed by a skull clamp in a slightly flexed, neutral position. A left frontal craniotomy was performed through a bicoronal incision, providing access to the anterior cranial fossa via a left interhemispheric approach. Microsurgical dissection was conducted under high magnification with intraoperative neuromonitoring, which remained stable throughout the procedure without any significant changes. Following exposure of the interhemispheric fissure, the lesion was visualized with its anterior fleshy component and posterior cystic portion. Tumor dissection was carried out following clear cleavage planes, with submission of samples for intraoperative and definitive histopathology. Working from posterior to anterior, the cystic portions of the tumor were first opened and emptied, which decompressed the lesion and created room to address the solid component. Once this had been removed in its entirety, the floor of the anterior fossa came into full view from above, and the erosion of the ethmoidal bone suspected on imaging became directly apparent. This small ethmoidal defect was repaired with a muscular patch, sealed with Evicel™ and reinforced with TachoSil™. Throughout the dissection, the left optic nerve, the optic chiasm, the terminal segment of the internal carotid artery, and the adjacent A2 segments of the anterior cerebral arteries were identified and kept under direct control. The left olfactory tract was found to be separable from the lesion and was anatomically preserved on the affected side.

**Figure 3:**
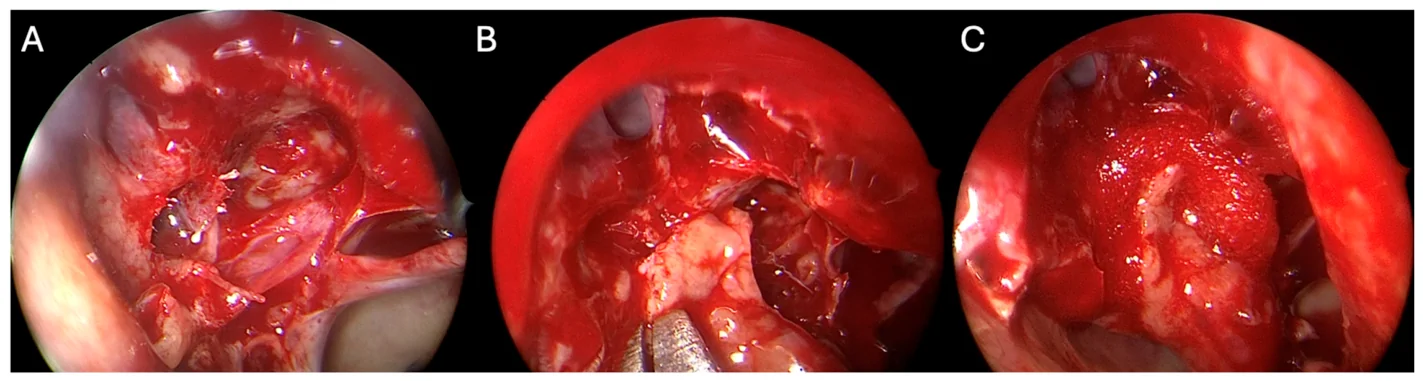
Endoscopic endonasal view. (**A**) Left ethmoid skull base showing a 5 mm medial defect. (**B**) Harvesting and positioning of a middle turbinate flap. (**C**) Final reconstruction with Floseal™ application.

To verify the integrity of the nasal mucosal lining, the rhinology team performed an endoscopic left ethmoidectomy. The skull base was remodeled and located at a much lower position. A 5 mm skull base defect was identified medially (Figure 3A). A middle turbinate flap was performed (Figure 3B) to add a nasal mucosal layer to the reconstruction, secured with Floseal™ (Figure 3C). The combined approach allowed this defect to be closed on both sides—intracranially from above and endonasally from below—so that, given its small size and low-flow nature, more extensive vascularized reconstructions such as a nasoseptal flap or fascia lata graft were not required.

### 2.3. Histopathological Findings

**Figure 4:**
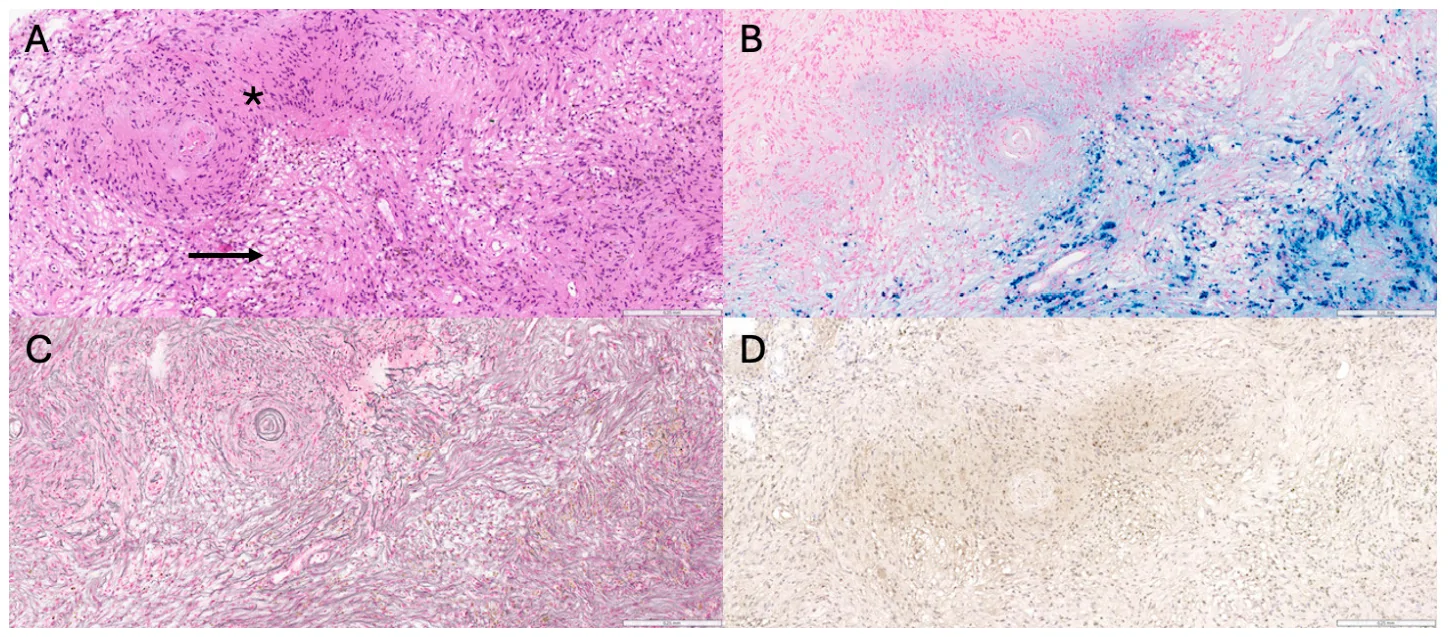
Histopathological examination showing features consistent with schwannoma. (**A**) Hematoxylin and eosin staining showing Antoni A (asterisk) and Antoni B (arrow) areas. (**B**) Prussian blue staining demonstrating hemosiderin deposits. (**C**) Gömöri reticulin stain highlighting the reticular framework within the tumor. (**D**) Strong S-100 protein immunopositivity confirming Schwann cell origin. Scale bar: 0.25 mm.

Histological evaluation revealed a biphasic spindle cell neoplasm composed of alternating Antoni A and Antoni B areas. Antoni A areas displayed elongated cells with eosinophilic cytoplasm, palisading nuclei, and Verocay bodies. Antoni B regions were looser, with microcystic architecture. Thick-walled hyalinized vessels and siderophages were observed. Immunohistochemistry showed strong positivity for S-100 and SOX10, consistent with Schwann cell origin. GFAP was negative within the tumor but positive in surrounding reactive astrocytes. The Ki-67 (MIB1) labeling index ranged from 1 to 8%. No expression of neurofilament (NF70) or epithelial membrane antigen (EMA) was detected. Perls’ Prussian blue staining highlighted hemosiderin deposits within siderophages, indicative of prior hemorrhage. These findings confirmed the diagnosis of WHO grade 1 schwannoma (Figure 4).

### 2.4. Postoperative Course and Follow-Up

**Figure 5:**
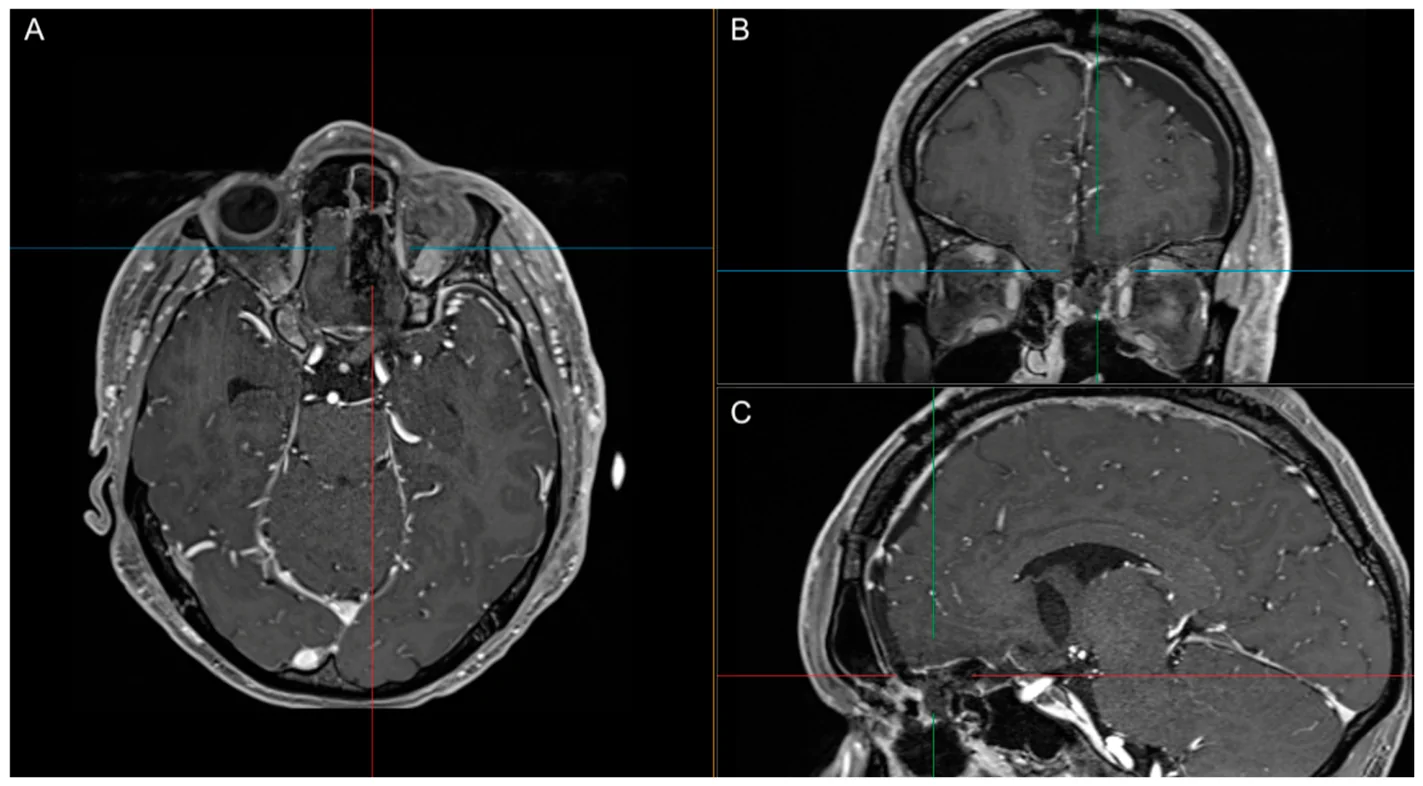
Postoperative T1 post-contrast MRI, showing complete resection of the lesion. (**A**) Axial T1 post-contrast image. (**B**) Coronal T1 post-contrast image. (**C**) Sagittal T1 post-contrast image.

The postoperative course was uneventful, with no new neurological deficits. The patient was discharged home on postoperative day 6, following confirmation of complete resection on postoperative MRI, observation for delayed CSF rhinorrhoea, and adequate healing of the nasal reconstruction. The main complications anticipated with this combined cranio-endonasal approach—CSF leak, pneumocephalus, meningitis, and nasal crusting—did not occur, and no worsening of olfactory or neurological function was observed (Figure 5).

Astonishingly, olfactory function improved at 1-month postoperatively on the left side from anosmia to hyposmia with a Sniffin’ Sticks TDI composite score of 20.5/48 (threshold = 1.5/16; discrimination = 6/16; identification 13//16) [13]. The right, unaffected side could be preserved (TDI = 34.5). Repeated measures at 9 months postoperatively confirmed this hyposmia which may be considered the left-sided final olfactory outcome (TDI = 18.5; T 1.5; D 7; I 10). This improvement is clinically meaningful and was sustained after 9 months [14]. There was no iatrogenic septal perforation that could explain this finding by biased contralateral olfactory perception. Postoperative neuropsychological and speech–language assessment showed restored lexical access and executive functioning, with naming performance within normal limits. A mild to moderate deficit in anterograde verbal memory persisted, particularly in delayed recall. Overall, the cognitive profile demonstrated favorable postoperative improvement.

The case was discussed at our institutional neuro-oncology tumor board. Given the complete excision and benign histology, a clinical and radiological surveillance strategy was adopted in which the patient will be followed with contrast-enhanced MRI every 6 months for at least 5 years. Neurosurgical and ENT evaluations will also take place at regular intervals.

## 3. Discussion

Olfactory groove schwannomas (OGSs) represent an exceptionally rare entity within the spectrum of intracranial nerve sheath tumors, accounting for less than 1% of all intracranial schwannomas [1]. Fewer than 80 cases have been documented in the literature, typically as isolated case reports or limited case series [2]. These tumors may originate from olfactory structures, such as the olfactory groove or cribriform plate, or from non-olfactory regions, as classified by Adachi et al. [15,16].

Given the absence of Schwann cells in the olfactory nerve, their origin remains a matter of debate [17]. Two prevailing theories have been proposed to explain their development [1,5,6,7,18]. The developmental hypothesis postulates that ectopic Schwann cells originate within the CNS due to aberrant migration of neural crest cells or metaplastic transformation of mesenchymal or pial cells. Alternatively, the non-developmental hypothesis attributes the origin of Schwann cells to perivascular autonomic plexuses, meningeal branches of the trigeminal nerve, or the olfactory ensheathing cells surrounding the olfactory axons, which share the same neural crest origin and also stain positive for S100.

Clinically, OGS may present with nonspecific symptoms such as headache, hyposmia or anosmia, and, less commonly, visual disturbances when mass effect involves the optic pathway [2,6,7]. In our case, the patient presented with transient visual disturbances over several months and a recent onset of intense headache. No signs of neurocognitive decline or personality changes were observed. He did not complain about subjective olfactory impairment, although he was found to be unilaterally anosmic. Unilateral anosmia, unlike other unilateral deafness or vision impairment, often stays clinically unrecognized. This is due to intact olfactory function on the right side and the mostly ipsilateral central wiring of olfactory paths. This highlights the importance of side-by-side smell testing in such cases because bilateral testing and self-ratings would have missed the finding of left-sided anosmia. For example, Li et al. performed a literature review and described 35 similar cases out of which 15 cases reported normal olfactory function. Most of these case reports did not report proper side-by-side smell testing [3,19,20,21,22]. In the present case, olfactory function improved on the affected side after tumor resection, a finding that has been reported previously but measured less precisely [9]. The confirmation of this counterintuitive improvement suggests a preoperative compression rather than direct involvement of the tumor and olfactory nerve. The latter may recover partly from pressure damage and uptake some function.

Other anterior skull base tumors may induce pressure damage of the olfactory system causing unilateral olfactory dysfunction, such as olfactory groove meningioma. Welge-Luessen and colleagues observed that four out of 12 patients had ipsilateral olfactory dysfunction with clear asymmetry in three of them, while four had ipsilateral normosmia and four had bilateral anosmia with large bilateral tumor. Preservation of the function ipsilateral to the tumor seems to be extremely difficult irrespective of the tumor size or surgical approach, and likehood of contralateral function preservation is high when the tumor is less than 3–4 cm in diameter.

The radiological appearance of OGS is variable and often misleading, posing a diagnostic challenge. On MRI, OGSs typically present as well-circumscribed, extra-axial masses with heterogeneous enhancement after gadolinium administration. They may contain both solid and cystic components and frequently exhibit mass effect on adjacent structures without significant peritumoral edema [1,7,10,12]. CT imaging can reveal remodeling or erosion of the underlying bone, especially when the lesion extends into the ethmoidal labyrinth or nasal cavity. These changes, however, are not pathognomonic and may resemble other anterior skull base tumors [23]. Our patient’s lesion demonstrated both solid and cystic components, displaced the optic chiasm and hypothalamus, and invaded the ethmoidal labyrinth with associated bone erosion. These features led to an initial radiological suspicion of esthesioneuroblastoma, underlining the importance of considering OGS in the differential diagnosis and highlighting the need to integrate imaging, histopathological, and immunohistochemical findings for accurate identification.

In this context, histopathology and immunoprofiling play a decisive role: these tumors typically display a biphasic pattern with Antoni A and B areas as well as Verocay bodies. Immunohistochemically, they show strong positivity for S100 protein and are negative for EMA, GFAP, and neuroendocrine markers, supporting their Schwann cell origin [3,6,24]. These characteristics are particularly relevant given the broad differential diagnosis of olfactory groove lesions, which includes esthesioneuroblastoma, meningioma, sinonasal carcinoma, and metastases [5,6,25]. Esthesioneuroblastoma is the most frequent radiologic mimic due to its location, bony erosion, and sinonasal extension. However, esthesioneuroblastomas typically show more aggressive features, such as a “dumbbell” shape across the cribriform plate, and express neuroendocrine markers (synaptophysin, chromogranin), unlike schwannomas [6,12]. Meningiomas may also appear well-circumscribed and enhancing but often show calcifications, hyperostosis, and dural tail and are EMA-positive [3,26]. Carcinomas and metastases are generally more infiltrative, poorly circumscribed, and lack the biphasic architecture and S-100 positivity typical of schwannoma [3,26].

Surgical resection remains the gold-standard treatment for OGS, with the choice of the surgical approach dictated primarily by the tumor’s location and degree of extension. Indeed, when the lesion is limited to the intracranial compartment, a single transcranial route—typically via a pterional or interhemispheric approach—is often sufficient to achieve gross total resection. The frontal interhemispheric approach, in particular, provides excellent exposure of the anterior cranial fossa while minimizing brain retraction [15,27]. However, when the tumor extends inferiorly into the ethmoidal labyrinth or nasal cavity, a combined approach becomes necessary. The transcranial route allows direct access to the anterior cranial fossa, enabling careful dissection around critical neurovascular structures such as the optic nerves and anterior cerebral arteries. Meanwhile, the endoscopic endonasal route provides safe and minimally invasive access to the sinonasal component, facilitates complete removal of the extracranial portion, and enables skull base reconstruction to avoid CSF leakage, adding an extra nasal mucosal layer to the skull base defect reconstruction [5,28]. This case illustrates that rationale: a purely endonasal route would not have controlled the intracranial neurovascular relationships or the high frontal cyst, whereas a purely transcranial route would not have addressed the sinonasal extension or allowed watertight repair of the ethmoidal defect from below.

Outcomes in the literature are generally favorable when gross total resection is achieved, and recurrence is rare. However, long-term follow-up remains essential [5,18]. We confirmed previously observed improvement of olfactory function in olfactory groove schwannoma postoperatively. Unlike esthesioneuroblastoma, which arises from the olfactory epithelium and whose resection typically compromises olfactory function on the operated side, our finding suggests secondary compression rather than primary involvement of the olfactory system. Olfactory testing is not a substitute for histopathology, which remains the definitive diagnostic modality. Rather, side-by-side assessment before and after surgery serves two distinct purposes: preoperatively, lateralized anosmia with contralateral normosmia supports the inclusion of olfactory groove schwannoma in the differential diagnosis and establishes a functional baseline; postoperatively, it documents preservation or improvement of olfaction, favoring secondary compression over primary involvement of the olfactory apparatus. In cases of suspected olfactory groove schwannoma it should thus always be kept in mind that besides mandatory tumor resection, olfactory preservation seems possible and may even be improved. We strongly emphasize the need to measure olfactory function pre- and preoperatively in a lateralized manner to unravel the full functional extent of smell function.

## Data Availability

The original contributions presented in this study are included in the article. Further inquiries can be directed to the corresponding authors.
